# Antagonisation of Prokineticin Receptor‐2 Attenuates Preeclampsia Symptoms

**DOI:** 10.1111/jcmm.70346

**Published:** 2025-01-16

**Authors:** Frédéric Sergent, Daniel Vaiman, Tiphaine Raia‐Barjat, Hadi Younes, Christel Marquette, Morgane Desseux, Roland Abi Nahed, Trinh‐Le‐Vi Kieu, Nguyen Viet Dung, Mathilde Keck, Pascale Hoffmann, Padma Murthi, Mohamed Benharouga, Nadia Alfaidy

**Affiliations:** ^1^ Interdisciplinary Research Institute of Grenoble, IRIG‐Biosanté University Grenoble Alpes, INSERM, CEA, UMR 1292 Grenoble France; ^2^ Commissariat à l'Energie Atomique et Aux Energies Alternatives (CEA) Biosciences and Biotechnology Institute of Grenoble Grenoble France; ^3^ Institute Cochin, U1016, INSERM, UMR 8504 CNRS, Paris‐Descartes Université Paris France; ^4^ Université Paris Saclay, CEA, INRAE, Département Médicaments et Technologies Pour la Santé (DMTS) Gif‐sur‐Yvette France; ^5^ Centre Hospitalo‐Universitaire Grenoble Alpes, Service Obstétrique, CS 10217 Grenoble France; ^6^ Université Grenoble Alpes Grenoble France; ^7^ Department of Pharmacology Monash Biomedicine Discovery Institute, Monash University Melbourne Victoria Australia; ^8^ Department of Maternal‐Fetal Medicine Pregnancy Research Centre The Royal Women's Hospital Melbourne Victoria Australia; ^9^ Department of Obstetrics and Gynecology The University of Melbourne Melbourne Victoria Australia

**Keywords:** preeclampsia, pregnancy, prokineticin 1 (PROK1), PROKR2, therapy

## Abstract

Preeclampsia (PE) is the most threatening pathology of human pregnancy. Placenta from PE patients releases harmful factors that contribute to the exacerbation of the disease. Among these factors is the prokineticin1 (PROK1) and its receptor, PROKR2 that we identified as a mediators of PE. Here we tested the effects of PKRA, an antagonist of PROKR2, on the attenuation of PE symptoms. We used the genetic PE mouse model, STOX1 that overexpresses *Stox1* gene in a heterozygosis manner in the placenta. This model allowed exploiting two genotypes of the offspring, those that overexpress the *Stox1* gene, and the WT that grow in a PE environment (STE). We characterised the effect PKRA (1 μM) on the attenuation of PE symptoms and compared its effects on STOX1 and STE placentas. We also used *STOX1* overexpressing trophoblast cells to decipher the PROK1‐underlying mechanism. We demonstrated that (i) antagonisation of PROKR2 attenuated PE‐mediated hypertension and proteinuria, (ii) STE placentas and foetuses exhibited better outcomes in response to PKRA, (iii) the secretome of STOX1‐trophoblasts impacted the integrity of the fetal vasculature that was attenuated by PKRA treatment. This study demonstrates the direct involvement of the PROK1 in PE and identifies PKRA as a promising therapy for PE.

## 1 Introduction

Preeclampsia (PE) is one of the most common and threatening pregnancy pathologies. It is associated with a high risk of feto‐maternal complications, such as fetal growth restriction (FGR), intra‐uterine death, preterm delivery, eclampsia, HELLP syndrome and other long‐term complications [1]. PE affects 2%–8% of all pregnancies and is classically defined by symptoms developing after 20 weeks of gestation (WG). These include high blood pressure (BP) > 140/90 mmHg, proteinuria and loss of the integrity of the vascular system [2]. PE has been reported to have both maternal and placental origins, however the underlying mechanisms are not fully understood [2]. Cumulative studies demonstrated that PE has also a genetic component [3], due to mutations and variants of the transcription factor STOX1 (Storkhead‐BOX Protein 1) [4, 5]. STOX1 overexpression in extravillous trophoblast cells (EVT) caused inhibition in their migration and invasion potential, two key processes that ensure normal placentation [6, 7]. In addition, transcriptomic analysis of first trimester placenta collected from pregnancies with PE showed elevated STOX1 gene expression (×2.1; *p* = 0.013) [8]. Importantly, STOX1 overexpression in the placenta of gravid WT mice triggered the development of early and severe PE symptoms (STOX1‐PE) [9]. This mouse model has successfully been used to test the efficacy of various drug treatments of PE, such as Aspirin, α1 Microglobulin, Tetrahydrobiotperin (BH4) [10, 11].

Despite significant progresses in the understanding of the causes of the development of PE, its complexity sustains many unanswered questions. It is well recognised that a failure in the process of EVT migration and invasion in the maternal decidua contributes to a poor placental perfusion and to the release by this organ of harmful anti‐angiogenic and inflammatory factors [1]. We have recently demonstrated that the placenta specific factor prokineticin1 (PROK1), also called EG‐VEGF (Endocrine Gland Derived Endothelial Growth Factor), exhibited features of a potential biomarker of pregnancy pathologies such as PE [12, 13, 14, 15, 16] and that its antagonization could be proposed as a therapeutic option in PE [12, 13, 14, 15, 16]. PROK1 is the canonical member of a recently described family of cytokines, the prokineticin family [17, 18]. Expression of PROK1 is essentially restricted to endocrine tissues, including the placenta [12, 13, 16, 19, 20]. It exerts its action via two G protein‐coupled prokineticin receptors, PROKR1 and PROKR2 [17, 18]. The latter is expressed by trophoblast and endothelial cells and has been reported to be increase in pathological settings [12, 13, 14, 15, 16].

Using the human placental explant model system, we demonstrated that PROK1 controls EVT invasion and endothelial cells integrity via PROKR2 [12, 13, 21]; that its circulating levels were 5‐fold higher during the first trimester of pregnancy [12, 13, 21] and were significantly increased in PE patients during the third trimester of pregnancy. These data strongly suggested that sustained PROK1 levels beyond the first trimester could contribute to the development of PE. By mimicking an increase in circulating PROK1 beyond 11.5 days post coitus (dpc) in the gravid mouse, we further provided evidence that the gravid mouse recapitulated key symptoms of PE including elevated BP [15]. Altogether, these studies suggested that antagonization of the PROKR2 may attenuate PROK1 adverse effects on the placenta and their consequences on the maternal and placental vascular system.

To further verify this hypothesis, we used the STOX1 transgenic mouse model of PE. First, we validated the up‐regulation of PROKR2 in the placentas of these mice and investigated the effect of the molecule PKRA, a non‐peptide PROKR2‐preferring antagonist [22]. Importantly, we recently provided evidence for the safety of PKRA use in normal gravid mice [23].

The model is based upon a cross between a heterozygous transgene carriers (the male mice) and WT females, resulting in litter half composed of WT animals and half of foetuses overexpressing *Stox1 gene*. This allowed characterising the effect of PROKR2 blockade on two types of placentas in the same uterus, STOX1 placentas overexpressing *Stox1* gene (~13 times more than the endogenous) and WT placentas that are not genetically modified, but grow in a PE environment. We surmise that these two types of placentas are representative of two conditions of PE. The first one where STOX1 overexpression caused the pathogenesis of PE that is triggered by placental failure (feto‐maternal origin), while the second one refers to a PE of maternal origin that affects the growth of a genetically normal placenta. To better understand the molecular influence of *Stox1‐*ovexpression in the placenta, we tested conditioned media from *Stox1‐*ovexpressing trophoblast cells on the integrity and migration of human placental endothelial cells, the HUVEC cells.

## 2 Material and Methods

### 2.1 In Vitro Study

#### 2.1.1 Cell Culture

The human trophoblast cell line JEG‐3 that overexpresses STOX1 gene was described previously [24]. The overexpressing cell line is called AA6 and the control JEG‐3 cells that were transfected by a control plasmid is called BD3 (CTL cells). For the rest of the article, AA6 will be called, JEG‐3‐STOX1. Cells were stably transfected with pCMX STOX1‐or with empty pCMX respectively [24]. Geneticin (G‐418, 500 μg/mL, Invitrogen) was continuously used to maintain the expression of the plasmid. JEG‐3‐STOX1 and CTL cells were maintained in Dulbecco's Modified Eagle Medium (DMEM) glutamine, containing 10% fetal bovine serum (FBS), 1% antibiotic–Penicillin/Streptomycin. Cells were incubated in a humidified 5% CO_2_ at 37°C. Both cell types were treated with vehicle (Ethanol 0.01%) or with the PROKR2 antagonist (PKRA, 1 μM, a gift from Dr. QY. Zhou) and incubated at 37°C. In another set of experiments, we tested the effect of JEG‐3‐STOX1 conditioned media (CM) on the integrity of the Human Umbilical Vein Endothelial Cells (HUVEC, ATCC).

Part of the methods involving analyses of trophoblast cells and endothelial cells (HUVEC cells) is reported in the Supporting Information S1.

### 2.2 In Vivo Study

#### 2.2.1 Animals

All animal studies were carried out in a strict accordance with the recommendations and the guidelines of the European Community for the Use of Experimental Animals (authorisation: 14176‐2018032016534632v1). The strain of mice used in this study is the FVB/N strain. The STOX1 transgenic line (called TgSTOX42) was previously described [9, 10]. This line was generated by introducing the human STOX1A ORF (complete isoform) under the control of a ubiquitously expressed promoter (the cytomegalovirus promoter) leading to a 13‐fold overexpression of the human STOX1 gene, relative to the endogeneous mouse gene Stox1 in the placenta. Female wild type mice FVB/N (WT) crossed with STOX42 male developed all symptoms of PE [9]. The date of the presence of a vaginal plug was considered as day 0.5 post coitus (dpc). All mice were housed under controlled illumination (12:12 h light:dark cycle). In the present study, we used four groups of mice. Group 1, called CTL, was composed of WT‐FVBN female mice (*n* = 8) crossed with WT‐FVB/N male mice (*n* = 8); Group 2, called STOX1, was composed of WT‐FVBN female mice (*n* = 8) crossed with TgSTOX42 male mice (*n* = 8); Group 3, called STOX1 + vehicle, was composed of WT‐FVBN female mice (*n* = 8) crossed with TgSTOX42 male mice (*n* = 8) and injected subcutaneously, with the vehicle of PKRA antagonist at days 7.5, 10.5 and 13.5 of gestation [23]; Group 4, called STOX1 + PKRA, was composed of WT‐FVBN female mice (*n* = 8) crossed with STOX42 male mice (*n* = 8) and injected subcutaneously with the PKRA antagonist (75 mg/kg) at days 7.5, 10.5 and 13.5 of gestation [23]. The groups 2, 3 and 4 produced litters overexpressing STOX1 from the paternal allele, heterozygote pups (50%), and WT pups (50%). Despite being genetically WT, the latter foetuses developed in a preeclamptic environment that we coined STOX‐environment (STE). Blood and urine collections were performed at day 15.5 of gestation (15.5 day post coïtus (dpc)), blood pressure (BP) measurements and treatment with PKRA was performed according to standardised protocols (see Flowchart, Figure 3). Gravid mice were sacrificed at 15.5 dpc. Maternal blood, placenta and foetuses were weighed and stored at −80°C or fixed for 24 h in 4% paraformaldehyde (PFA) for further analyses. Average weights were analysed as raw data. Placental efficiencies were determined by calculating the fetal/placental weight ratios.

### 2.3 Fetal and Placental Weights

Placentas and foetuses were weighted at 15.5 dpc and average weights were analysed as raw weights. Fetal and placental weights and fetal to placental weight ratio (reflecting placental efficiency) were compared between all groups [15].

### 2.4 Measurements of Blood Pressure and Assessment of Kidney Function

The mean blood pressures (BP) were measured using a noninvasive computerised tail‐cuff system (CODA 8 system, Paris, France) every day from day 0.5 to day 15.5 dpc. Data are represented as mean of arterial pressure values. Values of BP were grouped as follows: [0.5–7.5 dpc]; [8.5–10.5 dpc]; [11.5–13.5 dpc]; [14.5–15.5 dpc]. To assess kidney function, the mice were subjected to urine analysis. Urine was collected upon a passage of mice in metabolic chambers for 24 h between 14.5 and 15.5 dpc. Albumin and creatinine were measured in the urine specimens using the ELISA kit of Exocell (Philadelphia, PA).

### 2.5 Histological Analyses of the Placenta

For the histological analysis, placentas were collected at 15.5 dpc from WT, TgSTOX42 animals that were treated either with vehicle or with PKRA. Because of the heterozygosis of the placentas of the TgSTOX42 litter, both STOX1 and STE placentas were analysed.

We compared placentas collected from the following crossing: WT (FVBN male × FVBN female, *n* = 6); and TgSTOX42 (TgSTOX42 male × FVBN female, *n* = 6). The gravid mice were treated with vehicle or PKRA during their gestation (flowchart, Figure 4). We compared the following placentas: STOX1 + vehicle (*n* = 7), STE + vehicle (*n = 7*), STOX1 + PKRA (*n* = 7) and STE + PKRA (*n* = 7). Placental histology was performed as previously described [15]. Sections were stained with haematoxylin and eosin for general morphological analyses. The Axioplan (Zeiss) microscope and the Axiovision softaware were used to capture images. The surface of three placental zones (decidua, junctional zone, and labyrinth) were calculates using ImageJ software). Placental sections were also subjected to periodic acid‐Schiff (PAS) staining for the identification of glycogen cells.

### 2.6 Immunohistochemistry of Placental Tissues

Immunohistochemistry was performed as previously described [15]. Placental sections were incubated with the following antibodies, Anti‐CD31 (Abcam, 4 μg/mL, France), anti‐PCNA (proliferating cell nuclear antigen) (DAKO, Les Ulis, France). Immunopositive staining was detected using a Vectastain ABC kit (Vector Laboratories, Nanterre, France) and DAB (3, 3′‐diaminobenzidine tetrahydrochloride) as the chromogen (Vector Laboratories) [15].

### 2.7 Western Blotting of Placental Tissues

Western blotting analysis was performed as previously described [15]. Levels of expression of PROKR2, CD31 and PCNA were compared between WT, STE and STOX1 placentas. β‐actin was used to standardise for protein loading.

### 2.8 Statistical Analysis

Experiments were performed at least four times in an independent manner. Statistical comparisons were made using Student's *t*‐test and one way ANOVA. All data were checked for normality and equal variance. When normality failed, a nonparametric test followed by Dunn's or Bonferroni's test was used, (SigmaPlot and SigmaStat, Jandel Scientific Software). All data are expressed as means ± SE (***p ≤ 0.001), (**p ≤ 0.01), (*p ≤ 0.05).

## 3 Results

### 3.1 PROKR2 Expression in Placenta Collected From TgSTOX42 Gravid Mice

**FIGURE 1:**
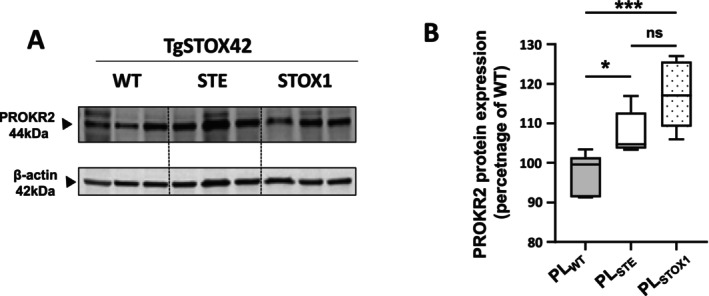
Comparison of the expression of PROKR2 in the WT, STE and STOX1 placentas. (A) reports a representative blot of the expression of STOX1 gene in WT, STE and STOX1 placentas collected from TgSTOX42 placentas. (B) depicts the quantification of PROKR2 protein levels in the same placentas. Data are expressed as mean ± SEM (*p < 0.05).

Breeding heterozygous TgSTOX42 male mice with WT female mice results in a litter composed of ½ STOX1 heterozygous placentas and ½ WT placentas that will develop in a PE environment [25]. We coined these placentas, STOX‐Environment (STE) while placentas carrying the transgene STOX1, are simply called STOX1. Western blot analyses of STE and STOX1 placentas showed that they both exhibit high levels of PROKR2, with greater levels observed in STOX1 placentas (Figure 1).

### 3.2 Validation of Hypertension and Renal Dysfunction in STOX1 Mice, TgSTOX42 Strain

**FIGURE 2:**
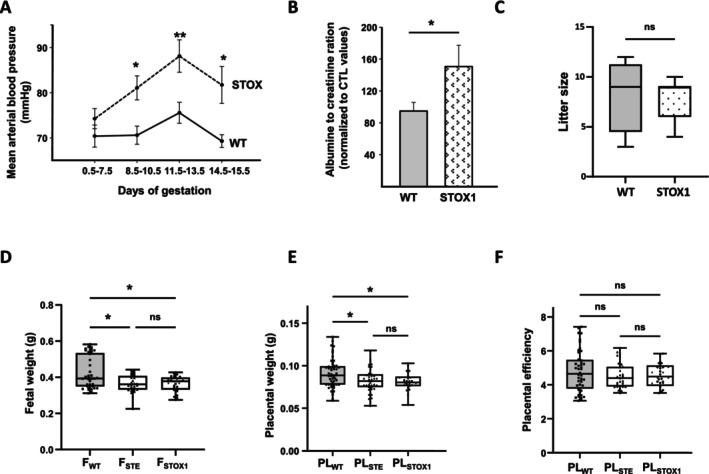
Characterisation of the TgSTOX42 pregnancy outcome. (A) reports a summary of the mean BP recorded longitudinally since day 0.5 until day 15.5 of gestation. Data are expressed as mean + SEM. (*p < 0.05, **p < 0.01). (B) reports a summary of 24‐h urinary Albumin/creatinine ratio normalised to control (CTL) values. Data are expressed as mean ± SEM. (*p < 0.05). (C) reports comparison of the litter size in WT and TgSTOX42 mice. Comparison of the number of STE and STOX1 foetuses in the litter size of TgSTOX42 mice is depicted in the underscore graph. (D) and (E) compare the fetal and placental weights in WT, STE and STOX1 specimens. (F) compares of the placental efficacy in the three types of placentas. Data are expressed as mean ± SEM (*p < 0.05).

The graph in Figure 2A reports a comparison of mean BP in control and STOX1 mice. BP was monitored daily, starting from the day of observation of the plug in the mated mice. An increase in BP during gestation was observed from 8.5 dpc (7.5–10.5). The curve of BP was significantly higher in STOX1 compared to CTL mice. The difference in BP was the highest at day 12.5 (11.5–13.5), still significant at day 13.5 (14.5–15.5), albeit much higher increase at 12.5 dpc. The maximal mean BP reached ≈78 mmHg in control mice and was ~15 mmHg more elevated in the STOX mice at 12.5 dpc.

We then assessed the renal function of CTL and STOX1 mice through the measurement of the albumin to creatinine ratio. Figure 2B depicts a significant increase in this ratio in the STOX1 mice, 1.8 fold, confirming the PE features of the model.

### 3.3 STE And STOX1 Placentas and Foetuses Exhibit Differential Growth Parameters

To address the effects of the PE on the progeny, we compared the litter size of CTL and STOX1 mice a trend to a decrease in the litter size in the STOX1 mice was observed; however, this did not reach significance (Figure 2C). Genotyping of mice within the STOX1 litters confirmed that the number of STE (genotypically WT) and STOX1 (genotypically positive for the transgene) foetuses was similar. However, we observed a significant decreases of the fetal weight of STE and STOX1 foetuses compared to WT mice (Figure 2D **)**. The weight of the placenta was significantly decreased in both phenotypes, albeit a stronger decrease was observed in the STOX placenta (Figure 2E). Placental efficiency did not change for both types of placentas (Figure 2H **)**.

### 3.4 STE and STOX1 Placenta are Structurally Distinct

Since we observed differences in weights of STE and STOX1 placentas, we analysed some of their features at the histological level, the labyrinth vascularization degree, the trophoblast proliferation and the invasion of glycogen cells.

**FIGURE 3:**
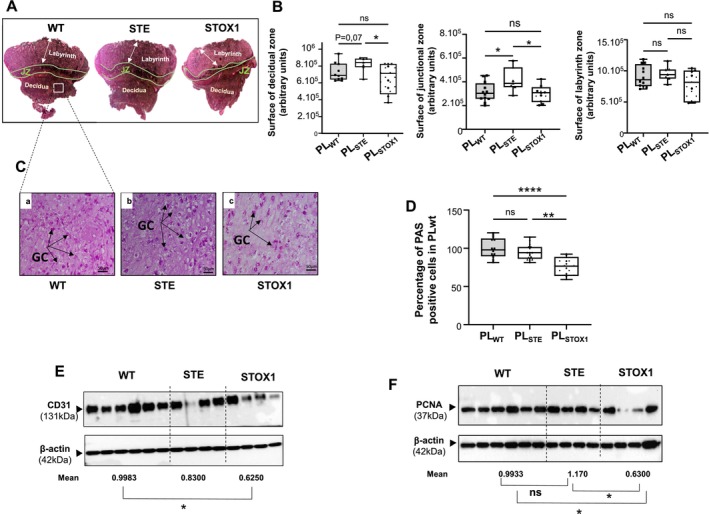
Analysis of the placental zones of WT, STE and STOX1 placentas and comparison of their vascularization and the proliferation and invasion of trophoblast cells. (A) Depicts representative placental section of WT, STE and STOX1 that were stained by Eosin–Haematoxylin. (B) shows graph that compare the surfaces of the three layers, the decidua, the junctional zone (JZ) and the labyrinth that were measured on parasagittal sections for each placenta. Data represent the mean ± SEM, (*p < 0.05). (C) and (D) Show three representative photographs of mouse WT, STE and STOX1 placental sections stained with Periodic Acid Schiff (PAS), purple colour, and the quantification of the percentage of PAS positive cells. (E) Shows a representative western blot that compares the levels of expression of CD31 protein in the three placentas. (F) Shows a representative western blot that compares the levels of expression of PCNA protein in the three placenta.

Basically, it is considered that each of the three placental zones (decidua, junctional zone (JZ), labyrinth) reflects specific aspects of its development. A decrease in the size of the decidual compartment zone reflects a lower degree of trophoblast invasion; an increase in the junctional zone is correlated with a higher degree of placental hypoxia, and the size of the labyrinth is informative on the degree of placental growth [26]. STOX1 placentas exhibited a significant decrease in the size of their decidual zone, no change in their junctional zone (JZ) and a trend to a decrease in their labyrinth zone, (Figure 3A,B). In contrast, the STE placenta showed a trend to an increase in the size of their decidual zone, a significant increase in their JZ and no change in their labyrinth zone. These data demonstrate differential developmental responses of STE and STOX1 placentas. While STOX1 placentas were intrinsically affected upon the overexpression of the STOX1 gene, the STE placenta phenotypes reflected the impact of a PE environment on the development of a WT placenta.

### 3.5 Trophoblast Invasion, Proliferation and Labyrinth Vascularization Are Decreased in STOX1 but Not in STE Placentas

Because trophoblast invasion leads to the establishment of the materno‐fetal circulation making the oxygenation of the placenta possible, we compared interstitial trophoblast invasion that is mainly mediated by glycogen cells in the rodents. Figure 3C depicts microphotographs of the representative WT, STE and STOX1 placentas stained for the glycogen cells. There was a significant decrease in the number of invasive trophoblast cells within the decidua of STOX1 placental tissues. However, no change was observed in the STE placentas (Figure 3C **)**.

The trend in the decrease in the weight of STOX1 placenta suggested a decrease in the branching and vascularization of their labyrinth and in the proliferation of trophoblast cells. Thus, we compared the level of expression of CD31, a marker of endothelial cells. Figure S1A depicts photomicrographs of CD31 staining in the labyrinth zone of WT, STE and STOX1 placentas. We observed less immunoreactivity for CD31 protein in STOX1 placentas compared to WT and STE. This was further confirmed by the quantitative comparison using Western blotting. Figure 3E depicts a representative Western Blot that compares CD31 levels in WT, STE and STOX placentas. There was a significant decrease in the expression of CD31 in STOX1 placentas. A trend to a decrease was observed in the STE placentas. We then compared the levels of the PCNA protein expression, a marker of cell proliferation. As for CD31 we observed a decrease in the PCNA staining in the labyrinth zone of the STOX1 (Figure S1B). Quantification of this expression using Western Blot analysis showed that PCNA levels were lower in STOX placenta and unchanged in STE placentas (Figure 3F).

### 3.6 Treatment With PKRA Attenuates Preeclampsia Symptoms in STOX1 Mice

Taking advantage from the STOX1 PE mouse model that offers the opportunity to study PE effects upon two different placental genotypes, we determined the effect of PKRA treatment on the outcome of the gestation and characterised placental changes following this treatment.

**FIGURE 4:**
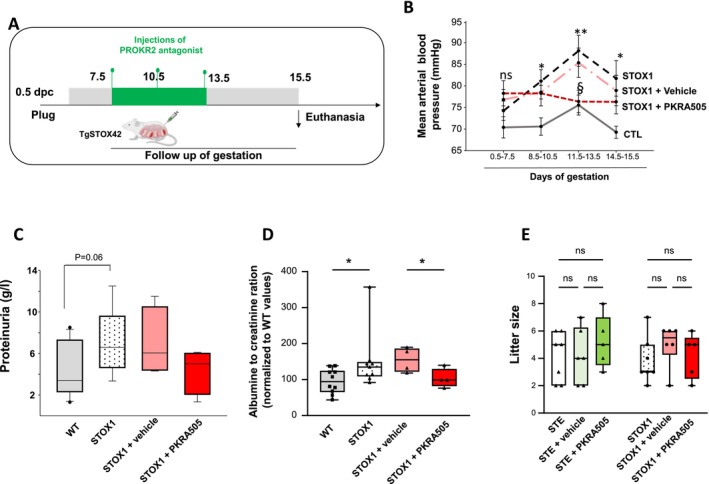
Effects of PKRA treatment on preeclampsia symptoms in the TgSTOX42 mouse model. (A) Illustrates the flow chart of the experimental procedure executed at different time‐points during gestation. The gravid mice TgSTOX42 were randomly assigned to be injected with either vehicle (*n* = 8 mice) or PROKR2 antagonist (PKRA) (*n* = 8 mice). The treatment with PKRA started on day 7.5 of gestation and was repeated every 3 days, at 10.5 dpc and at 13.5 dpc. Mice were sacrificed at 15.5 dpc. Placentas, foetuses, maternal kidney, urine and blood were collected. Blood pressure was measured for all animals since day 0.5 until day 15.5 of gestation. (B) Reports a summary of the mean BP recorded longitudinally since day 0.5 until day 15.5 of gestation. Data are expressed as mean ± SEM. (*p < 0.05, **p < 0.01). The graph recalls the summary of BP reported in Figure 2 and reports the evolution of the BP in mice treated with the vehicle (*n* = 8 mice) of PKRA (*n* = 8 mice) or PKRA. (C) Reports a comparison of proteinuria in urine collected in 24 h, in CTL, TgSTOX42, TgSTOX42 + vehicle and TgSTOX42 + PKRA. (D) Reports a comparison of urinary Albumin/creatinine values, in CTL, TgSTOX42, TgSTOX42 + vehicle and TgSTOX42 + PKRA. All values were normalised to control CTL. Data are expressed as mean ± SEM (*p < 0.05). (E) Reports a graph that compares the litter size of STE‐vehicle, STE‐PKRA, STOX1‐vehicle, STOX1‐PKRA.

Previous studies from our group demonstrated that the treatment of normal gravid mice with PKRA did not cause any detrimental effects to the outcome of gestation [23]. The flow chart depicted in Figure 4A reports the protocol used to treat the STOX1 (TgSTOX42) and WT mice with PKRA. The same concentrations and time points were used as previously reported [23].

To determine the effect of PROKR2 antagonists on the preeclampsia feature in STOX1 mice, a first group of pregnant mice (crossed with a transgenic male TgSTOX42) was treated with PKRA at three time points of gestation, 7.5, 10.5 and 13.5 dpc whilst a second group was treated with the vehicle at the three time points and considered as the control for the PKRA treatment. Figure 4B reports mean BP of STOX1 and CTL mice at four intervals of gestation. The graph also reported mean BP of STOX1 mice, treated three times with PKRA505 or vehicle. At the first period (0.5–7.5 days) no condition reaches significance. At 8.5–10.5 dpc, a significant increase of blood pressure was observed in STOX1 and STOX1 vehicle, compared to the CTL mice. At the blood pressure peak (11.5–13.5 period), the treatment with PKRA brought the blood pressure to the CTL levels, while the treatment with vehicle was not different. At the last period (14.5–15.5 days), the blood pressure under treatment was decreased but this did not reach significance. We then assessed the maternal renal function through the measurement of the maternal proteinuria and albumin to creatinine ratio. Figure 4C shows that the treatment of STOX1 mice with PKRA caused a trend to a decrease in the proteinuria compared to that of STOX1 mice and STOX1 treated with vehicle. When the proteinuria was corrected by creatinine levels (ACR ratio) the same result was observed (Figure 4D). These results indicate that the treatment with PKRA attenuates key symptoms of preeclampsia in the STOX1 model.

### 3.7 PKRA Treatment Ameliorates the Growth of STE but Not STOX1 Foetuses

In humans, PE is often associated with FGR (~1/3 of the cases), hence, we determined the potential benefit of PKRA treatment on fetal growth. Figure 4E shows that PKRA did not affect the litter size of all animals but tended to ameliorate the weights of the placentas and foetuses of STOX1 and STE. However, this improvement was only significant in the STE placentas and foetuses (Figure S2A,B, respectively). These results suggest that STE foetuses exhibited better response to PKRA treatment. No changes were observed in the efficiency of STE or STOX1 placentas (Figure 4D).

**FIGURE 5:**
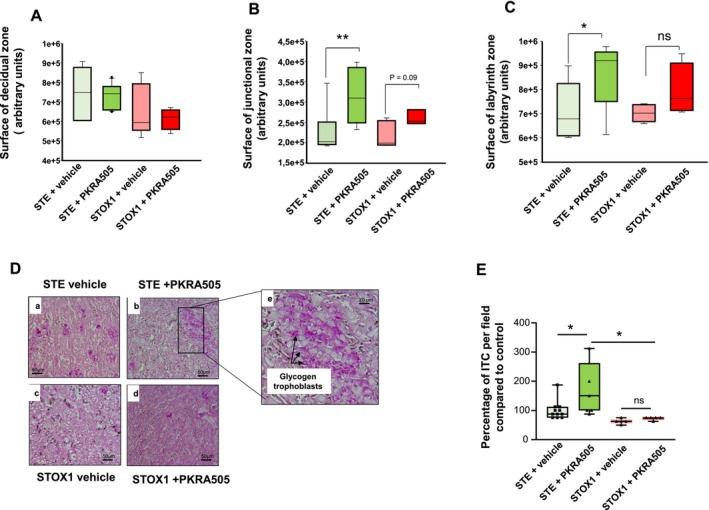
Effect of the treatment with PKRA on placental structure and trophoblast invasion. (A) Depicts a graph that compares the surface area of the decidual zone of STE‐vehicle, STE‐PKRA, STOX1‐vehicle and STOX1‐PKRA placentas. (B) Depicts a graph that compares the surface area of the junctional zone of STE‐vehicle, STE‐PKRA, STOX1‐vehicle and STOX1‐PKRA placentas. (C) Depicts a graph that compares the surface area of the labyrinth zone of STE‐vehicle, STE‐PKRA, STOX1‐vehicle and STOX1‐PKRA placentas. (D) Shows four representative photographs of STE‐vehicle, STE‐PKRA, STOX1‐vehicle, STOX1‐PKRA decidua stained with PAS, purple colour. Scale bar: 50 μm. (E) Presents the quantification of the invading cells as percentage of invasive trophoblast cells (ITC) per field compared to CTL condition. Data are expressed as mean + SEM (*p < 0.05).

Structural analyses of the placentas collected from STE and STOX1 groups demonstrated that PKRA induced a significant increase in the surface of the junctional and the labyrinth zones of the STE foetuses, while the surface of the decidua was not changed (Figure 5A–C). These changes were accompanied with an increase in the number of glycogen trophoblast cells that invaded the decidua of STE placentas (Figure 5D,E).

### 3.8 STOX1 Conditioned Media Impacted the Integrity of Fetal Vascular System

The above in vivo studies substantiated the former data obtained by Doridot and coworkers demonstrating that STOX1 overexpression in the placenta caused the development of preeclampsia and that the main symptoms could be attenuated upon treatment with aspirin [9]. The beneficial effect of aspirin was attributed to its effects on the maternal vascular system [11]. Here, we observed that the STE placenta and foetuses, that are genetically Wild Type, are strongly affected by the PE environment generated by the other foetuses. The origin of this PE environment can likely be found in the conditioned media (CM) of STOX1 overexpressing trophoblast cells. Hence, it was tempting to speculate that the PE‐STOX1 CM may not only affects the maternal vascular system but also the fetal vascular system. To verify this hypothesis, we cultured HUVEC and treated them by CM collected from STOX1‐overexpressing trophoblast cells, treated or not by PKRA.

### 3.9 STOX1 Trophoblast Overexpressing Cells Express High Levels of PROKR2 Receptor

Because microarray analysis of placenta collected from STOX1 mice presented with an increased expression of Prokr2 mRNA, which is reflected at the protein level as well, we compared the levels of expression of this receptor in CTL and STOX1 overexpressing trophoblast, the JEG‐3‐STOX1 cells. First, we validated that JEG‐3‐STOX1 overexpressed the STOX1 gene (Figure S3A,B) and demonstrated that the same cells overexpressed PROKR2 receptor (Figure S3C,D).

### 3.10 Treatment of JEG‐3‐STOX1 Cells With PKRA Attenuated the Impact of their CM on the Integrity of HUVEC

**FIGURE 6:**
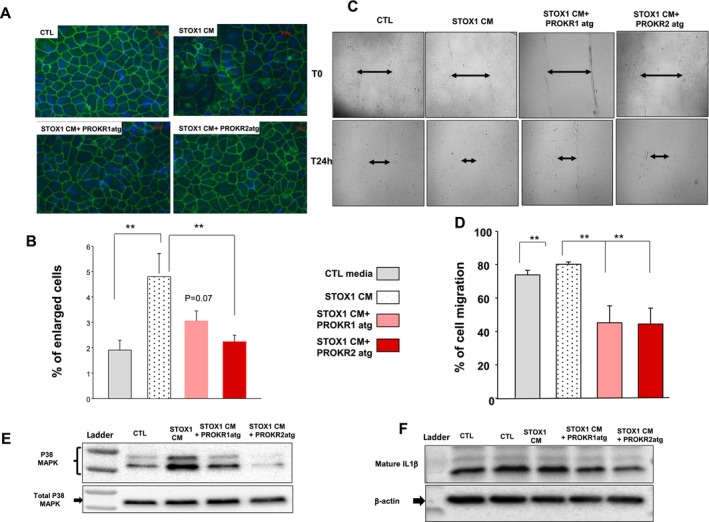
Effect of STOX1 conditioned media on HUVEC's integrity. (A) Reports representative photographs of HUVEC stained with VE‐Cadherin after 24 h of culture, in the absence (CTL) or presence of conditioned media (CM‐1/10 dilution) collected from STOX1 trophoblast cells (STOX1‐CM) that were treated with PKRA (1 μM) or PC7 (1 μM) for 24 h. (B) Reports a graph that compares the percentage of enlarged cells in each condition. (**p < 0.01). (C) Reports representative photographs of HUVEC imaged at T0 and 24 h after their wounding. HUVEC were incubated in the absence (CTL) or presence of CM (1/10 dilution) collected from STOX1 trophoblast cells (STOX1‐CM) that were treated with PKRA (1 μM) or PC7 (1 μM) for 24 h. The size of the wound was measured on photographs taken from three separate experiments using ImageJ. (D) Reports a graph that compares the percentage of cell migration in each condition. (**p < 0.01). (E) Reports a representative WB that compares the degree of phosphorylation of P38 MAPK in HUVEC cells that were incubated in the absence (CTL) or presence of CM (1/10 dilution) collected from STOX1 trophoblast treated with PKRA (1 μM) or PC7 (1 μM) for 24 h. (F) Reports a representative WB that compares the degree of maturation of IL‐1β in HUVEC that were incubated in the absence (CTL) or presence of CM (1/10 dilution) collected from STOX1 trophoblast cells treated with PKRA (1 μM) or PC7 (1 μM) for 24 h.

Previous study from Wang et al. [27] demonstrated that the permeability of endothelial cells was increased in response to unknown factors produced by PE trophoblast cells during their coculture with endothelial cells, suggesting that factors released from trophoblast cells of preeclamptic pregnancy, affect the endothelial cell barrier [27]. Here, we tested the effects of CM collected from STOX1 overexpressing cells on the subcellular localization of the major cell–cell adhesion molecule present at the endothelial adherent junctions, the VE‐cadherin [28]. Using immuno‐fluorescence, we showed that treatment of HUVEC with CM induced the formation of intercellular gaps in their monolayer as seen by a discontinuous VE‐cadherin staining at the periphery of the cells (Figure 6A). Compared with control, HUVEC treated with STOX1‐CM exhibited important morphological changes. The cells were larger and their shape was elongated and irregular. These morphological changes were significantly attenuated when cells were treated with CM collected from STOX1 overexpressing cells that were pretreated with PKRA (PROKR2 antagonist) at 1 μM (STOX‐PKRA‐CM). Because trophoblast cells also express PROKR1 receptor, we also tested a preferential antagonist of this receptor, the PC7 [23, 29]. Figure 6B shows the quantification of these observations. Overall, pre‐treatment with PKRA brought back the number of enlarged cell to the basal level.

### 3.11 Conditioned Media From STOX1 Trophoblasts Increased HUVEC's Migration

In order to migrate, endothelial cells should detach from neighbouring cells. To determine whether the effect of STOX1‐CM on HUVEC's integrity has any consequence on their migration, we compared the effect of the CTL, STOX1‐CM, STOX1‐CM‐PKRA, STOX1‐CM‐PC7 and media on the migration of HUVEC as shown in Figure 6C,D. CM from STOX1 cells increased HUVEC's migration while pretreatment of these cells with PKRA (1 μM) or PC7 (1 μM) reversed these effects.

### 3.12 STOX1 Conditioned Media Increases Inflammatory Pathways in HUVEC

Previous studies from the literature reported that the treatment of HUVEC cells with PE serum induced an oxidative stress, suggesting a dysregulation in the HUVEC's inflammatory responses [30]. We compared the levels of expression of the P38MAPK, a protein known to be increased under stressed and inflammatory conditions in HUVEC. Figure 6E show that CM from STOX1 cells induced the phosphorylation of P38MAPK and that this effect was attenuated when HUVEC were pre‐incubated with CM collected from STOX1‐PKRA‐CM or STOX1‐PC7‐CM.

To determine whether the CM mediated‐inflammatory effect was accompanied with the expression of inflammatory cytokines such as IL1‐β, we compared the levels of expression of the mature form of IL‐β in all conditions mentioned above. Figure 6F shows that HUVEC treated with CM from STOX1 cells expressed higher levels of IL‐1β and that this effect was decreased in the presence of the antagonist PKRA. Because the increase in the levels of IL‐1β is tightly dependent on the intracellular production of pro‐IL‐1β that can be produced upon the activation of the inflammasome machinery, we compared the expression of key proteins of the inflammasome machinery, *ie*: caspase‐1, the adaptor molecule apoptosis‐associated speck‐like protein containing a CARD (ASC) and the pro‐ILβ and Il‐1β. Figure S4A–D show that STOX1 overexpressing cells express higher levels of NLRP7, ASC, caspase‐1, and pro‐Il‐1β and IL‐1β, respectively. These data strongly suggest that overexpression of *Stox1* directly contributes to the activation of the inflammasome pathway in trophoblast cells.

## 4 Discussion

The present study demonstrates that preeclampsia‐adverse effects observed in the genetic model STOX1 were attenuated upon the treatment of the STOX1‐gravid mice with the antagonist of PROKR2 receptor, the PKRA. This antagonist has been shown to efficiently inhibit PROKR2 receptor with an IC50 of 48.1 ± 4.6 nM [22]. The choice of PKRA as a potential treatment of PE was based on previous studies from our group demonstrating that PROKR2 was directly involved in the regulation of PROK1‐mediated trophoblast migration and invasion, and endothelial cell's integrity [21, 31, 32]. Importantly, we recently brought evidence for the safety and effectiveness of the use of PKRA during pregnancy [23]. The present proof of concept uncovered the endogenous functions of PROK1 and demonstrated that the use of the antagonist of its receptor PROKR2 enhances the feto‐placental growth and ameliorated the pregnancy outcome [23]. The safe features of PROKR2 antagonists were expected as it was reported in numerous studies to exhibit both analgesic and anti‐inflammatory effects [33].

The in vitro study that used overexpressing‐*Stox1* trophoblast cells brought further evidence that the blockade of PROKR2 attenuated STOX1‐mediated adverse effects, including the restoration of the PE mediated endothelial cell's disorganisation. The blockade of PROKR2 also lessened endothelial cell's inflammation through the decrease of the phosphorylation of p38‐MAP Kinase and maturation of IL‐1β, likely to originate from several intracellular sources, including the activation of the inflammasome.

The attenuation of endothelial cells disorganisation in response to CM of PKRA‐pretreated trophoblast cells strongly suggests that PROKR2 signalization may directly be involved in the cross talk between trophoblast and endothelial cells. Importantly, we demonstrated that endothelial cells also express PROKR2 [31]. Hence, one can speculate that when used in vivo, PKRA may not only act on trophoblast cells but also on endothelial cells. This statement is in line with studies from the group of Nebgil who demonstrated that PROKR2 overexpressing endothelial cells display a larger number of multivesicular bodies and caveolar clusters along with a disruption of the distribution of zonula occluden‐1 tight junction (ZO‐1) proteins [34, 35]. To implement this study, it would be interesting to determine the direct effect of PKRA on endothelial cells collected from PE women. This may strengthen the consideration of PKRA as a novel therapeutic option in PE women.

The preeclampsia mouse model used in the in vivo study substantiated the evidence that overexpression of the *Stox1* gene in the placenta induces the development of PE symptoms and brought new evidence of the direct influence of the STOX1 on the pregnancy outcome. Overexpression of STOX1 in the placenta not only impacted the STOX1 placentas but also created an environment that impacted the growth of WT placentas and foetuses that evolve in the same uterine horns (STE). These statements are based on several key findings. First, the STOX1 placentas were more affected in terms of growth, structural organisation, vascularization and trophoblast invasion compared to STE placentas. The latter exhibited fewer disorganizations, likely attributed to compensatory mechanisms known to occur in response to any PE environment [36, 37]. The minor effects observed on the WT (STE) placentas may explain some of the features that a normal placenta may experience while growing in a PE environment of maternal origin. Second, STE placentas have a significant increase in their junctional zone, a sign of placentas suffering from a hypoxic environment, also a feature of PE of maternal origin [38]. Third, the CM of STOX1 trophoblast cells affected the integrity of fetal endothelial cells. This result strongly suggests that STOX1 trophoblast cells may secrete several factors in the intervillous space that may not only influence neighbouring trophoblast cells but also maternal and fetal vascular systems [39, 40].

The demonstration that PROKR2 was significantly increased in STOX1 placentas and in trophoblast cells overexpressing this gene, suggested that PROKR2 could transcriptionally be regulated by the STOX1 factor, however, a significant increase in the expression of this gene was also observed in the STE placentas that does not overexpress the *STOX1* gene. This finding suggests that PROKR2 increased expression might also occur in an independent regulatory pathway of STOX1. Hence, one can speculate that PROKR2 increased expression may also occur in normal placentas growing in PE of maternal origin. In line with this statement is the demonstration that STE placentas exhibited better responses to PKRA treatment, especially in terms of fetal and placental growth, that was confirmed by the increase in the size of their labyrinth zone and their invasion of their trophoblasts, the two key features of the occurrence of compensatory mechanisms that allow the progression of pregnancy.

## Author Contributions

**Frédéric Sergent:** investigation (equal), methodology (equal). **Daniel Vaiman:** data curation (lead), investigation (supporting), writing – original draft (supporting). **Tiphaine Raia‐Barjat:** funding acquisition (equal), investigation (equal), methodology (equal), writing – review and editing (equal). **Hadi Younes:** investigation (supporting), methodology (supporting). **Christel Marquette:** methodology (supporting). **Morgane Desseux:** data curation (lead), methodology (supporting). **Roland Abi Nahed:** data curation (lead), investigation (supporting), methodology (supporting). **Trinh‐Le‐Vi Kieu:** investigation (supporting), methodology (lead), visualization (supporting). **Nguyen Viet Dung:** data curation (supporting), investigation (supporting), methodology (supporting). **Mathilde Keck:** investigation (supporting), visualization (supporting), writing – original draft (supporting). **Pascale Hoffmann:** conceptualization (supporting), investigation (supporting), writing – original draft (supporting). **Padma Murthi:** supervision (supporting), writing – original draft (equal), writing – review and editing (equal). **Mohamed Benharouga:** conceptualization (lead), funding acquisition (lead), writing – review and editing (supporting). **Nadia Alfaidy:** conceptualization (equal), formal analysis (equal), investigation (equal), methodology (equal), project administration (equal), resources (equal), supervision (equal), validation (equal), writing – review and editing (equal).

## Ethics Statement

All animal studies were carried out in a strict accordance with the recommendations and the guidelines of the European Community for the Use of Experimental Animals (authorisation: 14176‐2018032016534632v1).

## Conflicts of Interest

The authors declare no conflicts of interest.

## Supporting information


Data S1.

## Data Availability

The data that support the findings of this study are available from the corresponding author upon reasonable request.

## References

[1] B. Sibai , G. Dekker , and M. Kupferminc , “Pre‐Eclampsia,” Lancet 365 (2005): 785–799. 15733721 10.1016/S0140-6736(05)17987-2

[2] E. Dimitriadis , D. L. Rolnik , W. Zhou , et al., “Pre‐Eclampsia,” Nature Reviews Disease Primers 9 (2023): 8. 10.1038/s41572-023-00417-6 36797292

[3] S. Cnattingius , M. Reilly , Y. Pawitan , and P. Lichtenstein , “Maternal and Fetal Genetic Factors Account for Most of Familial Aggregation of Preeclampsia: A Population‐Based Swedish Cohort Study,” American Journal of Medical Genetics Part A 130A (2004): 365–371. 15384082 10.1002/ajmg.a.30257

[4] A. S. Jimenez‐Osorio , E. Carreon‐Torres , E. Correa‐Solis , et al., “Inflammation and Oxidative Stress Induced by Obesity, Gestational Diabetes, and Preeclampsia in Pregnancy: Role of High‐Density Lipoproteins as Vectors for Bioactive Compounds,” Antioxidants 12 (2023): 12. 10.3390/antiox12101894 PMC10604737 37891973

[5] M. van Dijk , J. Mulders , A. Poutsma , et al., “Maternal Segregation of the Dutch Preeclampsia Locus at 10q22 With a New Member of the Winged Helix Gene Family,” Nature Genetics 37 (2005): 514–519. 15806103 10.1038/ng1541

[6] C. E. Dunk , M. van Dijk , R. Choudhury , et al., “Functional Evaluation of STOX1 (STORKHEAD‐BOX PROTEIN 1) in Placentation, Preeclampsia, and Preterm Birth,” Hypertension 77 (2021): 475–490. 33356399 10.1161/HYPERTENSIONAHA.120.15619

[7] M. van Dijk , J. van Bezu , D. van Abel , et al., “The STOX1 Genotype Associated With Pre‐Eclampsia Leads to a Reduction of Trophoblast Invasion by Alpha‐T‐Catenin Upregulation,” Human Molecular Genetics 19 (2010): 2658–2667. 20400461 10.1093/hmg/ddq152

[8] S. A. Founds , Y. P. Conley , J. F. Lyons‐Weiler , A. Jeyabalan , W. A. Hogge , and K. P. Conrad , “Altered Global Gene Expression in First Trimester Placentas of Women Destined to Develop Preeclampsia,” Placenta 30 (2009): 15–24. 19027158 10.1016/j.placenta.2008.09.015 PMC2667803

[9] L. Doridot , B. Passet , C. Mehats , et al., “Preeclampsia‐Like Symptoms Induced in Mice by Fetoplacental Expression of STOX1 Are Reversed by Aspirin Treatment,” Hypertension 61 (2013): 662–668. 23357179 10.1161/HYPERTENSIONAHA.111.202994

[10] L. Chatre , A. Ducat , F. T. Spradley , et al., “Increased NOS Coupling by the Metabolite Tetrahydrobiopterin (BH4) Reduces Preeclampsia/IUGR Consequences,” Redox Biology 55 (2022): 102406. 35964341 10.1016/j.redox.2022.102406 PMC9389306

[11] A. Ducat , A. Vargas , L. Doridot , et al., “Low‐Dose Aspirin Protective Effects Are Correlated With Deregulation of HNF Factor Expression in the Preeclamptic Placentas From Mice and Humans,” Cell Death Discovery 5 (2019): 94. 31098302 10.1038/s41420-019-0170-x PMC6510804

[12] S. Brouillet , P. Hoffmann , J. J. Feige , and N. Alfaidy , “EG‐VEGF: A Key Endocrine Factor in Placental Development,” Trends in Endocrinology and Metabolism 23 (2012): 501–508. 22709436 10.1016/j.tem.2012.05.006

[13] P. Hoffmann , J. J. Feige , and N. Alfaidy , “Expression and Oxygen Regulation of Endocrine Gland‐Derived Vascular Endothelial Growth Factor/Prokineticin‐1 and Its Receptors in Human Placenta During Early Pregnancy,” Endocrinology 147 (2006): 1675–1684. 16384869 10.1210/en.2005-0912

[14] D. Reynaud , F. Sergent , R. Abi Nahed , S. Brouillet , M. Benharouga , and N. Alfaidy , “EG‐VEGF Maintenance Over Early Gestation to Develop a Pregnancy‐Induced Hypertensive Animal Model,” Methods in Molecular Biology 1710 (2018): 317–324. 29197014 10.1007/978-1-4939-7498-6_25

[15] F. Sergent , P. Hoffmann , S. Brouillet , et al., “Sustained Endocrine Gland‐Derived Vascular Endothelial Growth Factor Levels Beyond the First Trimester of Pregnancy Display Phenotypic and Functional Changes Associated With the Pathogenesis of Pregnancy‐Induced Hypertension,” Hypertension 68 (2016): 148–156. 27141059 10.1161/HYPERTENSIONAHA.116.07442

[16] W. Traboulsi , S. Brouillet , F. Sergent , et al., “Prokineticins in Central and Peripheral Control of Human Reproduction,” Hormone Molecular Biology and Clinical Investigation 24 (2015): 73–81. 26574895 10.1515/hmbci-2015-0040

[17] J. LeCouter , J. Kowalski , J. Foster , et al., “Identification of an Angiogenic Mitogen Selective for Endocrine Gland Endothelium,” Nature 412 (2001): 877–884. 11528470 10.1038/35091000

[18] D. C. Lin , C. M. Bullock , F. J. Ehlert , J. L. Chen , H. Tian , and Q. Y. Zhou , “Identification and Molecular Characterization of Two Closely Related G Protein‐Coupled Receptors Activated by Prokineticins/Endocrine Gland Vascular Endothelial Growth Factor,” Journal of Biological Chemistry 277 (2002): 19276–19280. 11886876 10.1074/jbc.M202139200

[19] N. Alfaidy , “Prokineticin1 and Pregnancy,” Annales d'Endocrinologie 77 (2016): 101–104. 10.1016/j.ando.2016.04.014 27172869

[20] P. Hoffmann , J. J. Feige , and N. Alfaidy , “Placental Expression of EG‐VEGF and Its Receptors PKR1 (Prokineticin Receptor‐1) and PKR2 Throughout Mouse Gestation,” Placenta 28 (2007): 1049–1058. 17531315 10.1016/j.placenta.2007.03.008

[21] P. Hoffmann , Y. Saoudi , M. Benharouga , et al., “Role of EG‐VEGF in Human Placentation: Physiological and Pathological Implications,” Journal of Cellular and Molecular Medicine 13 (2009): 2224–2235. 19602057 10.1111/j.1582-4934.2008.00554.x PMC6512391

[22] M. Y. Cheng , A. G. Lee , C. Culbertson , et al., “Prokineticin 2 Is an Endangering Mediator of Cerebral Ischemic Injury,” Proceedings of the National Academy of Sciences of the United States of America 109 (2012): 5475–5480. 22431614 10.1073/pnas.1113363109 PMC3325724

[23] D. Reynaud , F. Sergent , R. Abi Nahed , et al., “Evidence‐Based View of Safety and Effectiveness of Prokineticin Receptors Antagonists During Pregnancy,” Biomedicine 9 (2021): 9. 10.3390/biomedicines9030309 PMC8002561 33802771

[24] V. Rigourd , C. Chauvet , S. T. Chelbi , et al., “STOX1 Overexpression in Choriocarcinoma Cells Mimics Transcriptional Alterations Observed in Preeclamptic Placentas,” PLoS One 3 (2008): e3905. 19079545 10.1371/journal.pone.0003905 PMC2592700

[25] G. J. Burton , C. W. Redman , J. M. Roberts , and A. Moffett , “Pre‐Eclampsia: Pathophysiology and Clinical Implications,” BMJ 366 (2019): l2381. 31307997 10.1136/bmj.l2381

[26] J. C. Cross , D. Baczyk , N. Dobric , et al., “Genes, Development and Evolution of the Placenta,” Placenta 24 (2003): 123–130. 12596737 10.1053/plac.2002.0887

[27] Y. Wang , D. F. Lewis , Y. Gu , Y. Zhang , J. S. Alexander , and D. N. Granger , “Placental Trophoblast‐Derived Factors Diminish Endothelial Barrier Function,” Journal of Clinical Endocrinology and Metabolism 89 (2004): 2421–2428. 15126573 10.1210/jc.2003-031707

[28] E. Dejana and F. Orsenigo , “Endothelial Adherens Junctions at a Glance,” Journal of Cell Science 126 (2013): 2545–2549. 23781019 10.1242/jcs.124529

[29] E. Landucci , R. Lattanzi , E. Gerace , et al., “Prokineticins Are Neuroprotective in Models of Cerebral Ischemia and Ischemic Tolerance In Vitro,” Neuropharmacology 108 (2016): 39–48. 27140692 10.1016/j.neuropharm.2016.04.043

[30] Z. Q. Qian , X. P. Li , C. Z. Wang , X. Y. He , and F. Fang , “Oxidative Damage Effect of the Serum of Severe Preeclamptic Patients on Human Umbilical Vein Endothelial Cell,” Zhonghua Fu Chan Ke Za Zhi 48 (2013): 193–197. 23849942

[31] S. Brouillet , P. Hoffmann , M. Benharouga , et al., “Molecular Characterization of EG‐VEGF‐Mediated Angiogenesis: Differential Effects on Microvascular and Macrovascular Endothelial Cells,” Molecular Biology of the Cell 21 (2010): 2832–2843. 20587779 10.1091/mbc.E10-01-0059 PMC2921113

[32] V. Garnier , W. Traboulsi , A. Salomon , et al., “PPARgamma Controls Pregnancy Outcome Through Activation of EG‐VEGF: New Insights Into the Mechanism of Placental Development,” American Journal of Physiology. Endocrinology and Metabolism 309 (2015): E357–E369. 26081281 10.1152/ajpendo.00093.2015

[33] F. Guida , R. Lattanzi , S. Boccella , et al., “PC1, a Non‐peptide PKR1‐Preferring Antagonist, Reduces Pain Behavior and Spinal Neuronal Sensitization in Neuropathic Mice,” Pharmacological Research 91 (2015): 36–46. 25434589 10.1016/j.phrs.2014.11.004

[34] C. Guilini , K. Urayama , G. Turkeri , et al., “Divergent Roles of Prokineticin Receptors in the Endothelial Cells: Angiogenesis and Fenestration,” American Journal of Physiology Heart and Circulatory Physiology 298 (2010): H844–H852. 20023120 10.1152/ajpheart.00898.2009

[35] K. Urayama , D. B. Dedeoglu , C. Guilini , et al., “Transgenic Myocardial Overexpression of Prokineticin Receptor‐2 (GPR73b) Induces Hypertrophy and Capillary Vessel Leakage,” Cardiovascular Research 81 (2009): 28–37. 18806277 10.1093/cvr/cvn251

[36] L. Myatt , R. G. Clifton , J. M. Roberts , et al., “Can Changes in Angiogenic Biomarkers Between the First and Second Trimesters of Pregnancy Predict Development of Pre‐Eclampsia in a Low‐Risk Nulliparous Patient Population?,” BJOG: An International Journal of Obstetrics and Gynaecology 120 (2013): 1183–1191. 23331974 10.1111/1471-0528.12128 PMC4104359

[37] L. Myatt and R. P. Webster , “Vascular Biology of Preeclampsia,” Journal of Thrombosis and Haemostasis: JTH 7 (2009): 375–384. 19087223 10.1111/j.1538-7836.2008.03259.x

[38] A. Wang , S. Rana , and S. A. Karumanchi , “Preeclampsia: The Role of Angiogenic Factors in Its Pathogenesis,” Physiology 24 (2009): 147–158. 19509125 10.1152/physiol.00043.2008

[39] M. H. Schoots , S. J. Gordijn , S. A. Scherjon , H. van Goor , and J. L. Hillebrands , “Oxidative Stress in Placental Pathology,” Placenta 69 (2018): 153–161. 29622278 10.1016/j.placenta.2018.03.003

[40] M. Zygmunt , F. Herr , K. Munstedt , U. Lang , and O. D. Liang , “Angiogenesis and Vasculogenesis in Pregnancy,” European Journal of Obstetrics, Gynecology, and Reproductive Biology 110, no. Suppl 1 (2003): S10–S18. 12965086 10.1016/s0301-2115(03)00168-4

