# Supplementary material for: Antagonisation of Prokineticin Receptor‐2 Attenuates Preeclampsia Symptoms
Source: J Cell Mol Med. 2025 Jan 16;29(2):e70346. doi: 10.1111/jcmm.70346 (PMC11736873; doi:10.1111/jcmm.70346)
Supplement: Supplementary file 1 — Data S1. [file JCMM-29-e70346-s001.docx]

*Supplementary figures and part of Material and methods*

**Antagonisation of prokineticin receptor-2 attenuates Preeclampsia symptoms**

Frédéric Sergent^1§^, Daniel Vaiman^2§^, Tiphaine Raia-Barjat^1^, Hadi Younes^1^, Christel Marquette^1^, Morgane Desseux^1^, Roland Abi Nahed^1^, Trinh-Le-Vi Kieu^1^, Nguyen Viet Dung^1^, Pascale Hoffmann^1,4^, Padma Murthi^5,6,7^, Mohamed Benharouga^1^*, and Nadia Alfaidy^1,4^*.

1. Interdisciplinary Research Institute of Grenoble, IRIG-Biosanté, University Grenoble Alpes, INSERM, CEA, UMR 1292, F-38000 Grenoble, France Commissariat à l’Energie Atomique et aux Energies Alternatives (CEA), Biosciences and Biotechnology Institute of Grenoble, Grenoble, France
2. Institute Cochin, U1016, INSERM, UMR 8504 CNRS, Paris-Descartes Université, 75014 Paris, France.
3. Université Paris Saclay, CEA, INRAE, Département Médicaments et Technologies pour la Santé (DMTS), SIMoS, 91191 Gif-sur-Yvette, France.
4. Centre Hospitalo-Universitaire Grenoble Alpes, Service Obstétrique, CS 10217, 38043 Grenoble Cedex 9, France ; Université Grenoble Alpes, France.
5. Department of Pharmacology, Monash Biomedicine Discovery Institute, Monash University, Melbourne, VIC 3800, Australia.
6. Department of Maternal-Fetal Medicine Pregnancy Research Centre, the Royal Women's Hospital, Melbourne, VIC 3052, Australia.
7. Department of Obstetrics and Gynecology, the University of Melbourne, Melbourne, VIC 3052, Australia.

*equal last authors

§equal first authors

Corresponding author: Nadia Alfaidy

ORCID: 0000-0002-0718-2447

Unité INSERM. Laboratoire Biosanté, U1292

CEA Grenoble 17, rue des Martyrs

38054 Grenoble cedex 9

Tel. 04 38 78 04 07, Fax 04 38 78 50 58

[nadia.alfaidy-benharouga@cea.fr](mailto:nadia.alfaidy-benharouga@cea.fr)

**Fig. Supp-1**


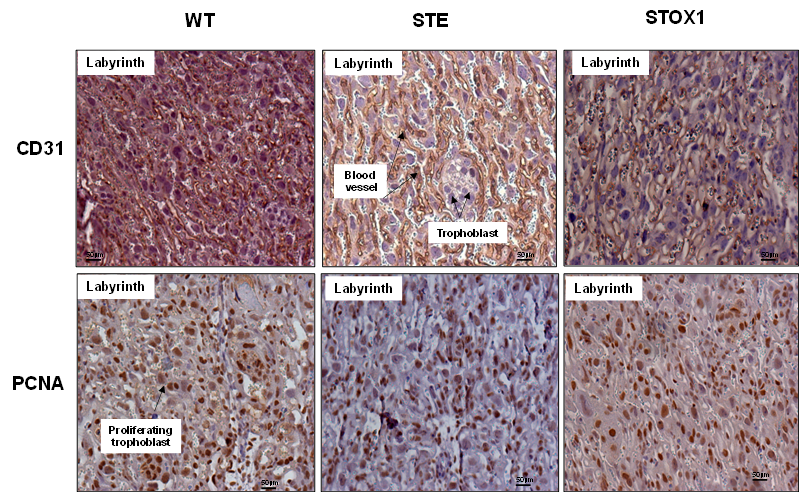


**Figure Supp-1: Comparison of the vascularization and proliferation of the trophoblasts in the labyrinth zone of placentas. Panel A** depicts representative placental sections of WT, STE and STOX1 that were stained by CD31 antibody. **Panel B** depicts representative placental section of WT, STE and STOX1 that were stained with PCNA antibody. Barr scale= 50 µm

**Fig. Supp-2**


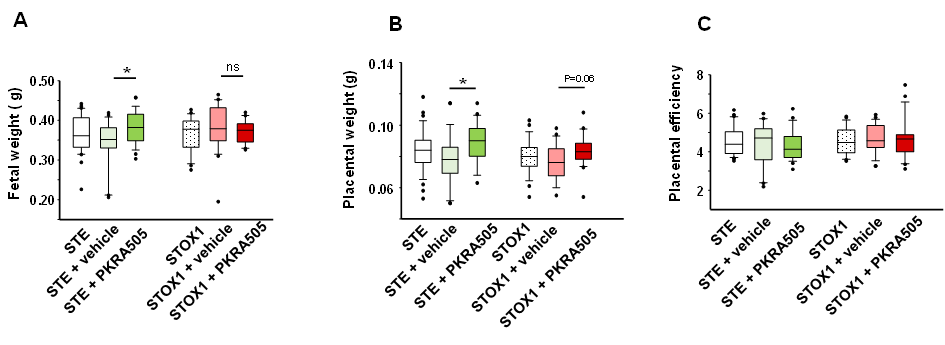


**Figure Supp-2. Effect of the treatment with PKRA on pregnancy outcome. Panel A** reports a graph that compares the fetal weight of STE-vehicle, STE-PKRA, STOX1-vehicle and STOX1-PKRA. **Panel B** reports a graph that compares the placental weight of STE-vehicle, STE-PKRA, STOX1-vehicle and STOX1-PKRA. **Panel C** compares the placental efficacy of STE-vehicle, STE-PKRA, STOX1-vehicle and STOX1-PKRA. Data are expressed as mean + SEM (*p < 0.05).

**Fig. Supp-3**


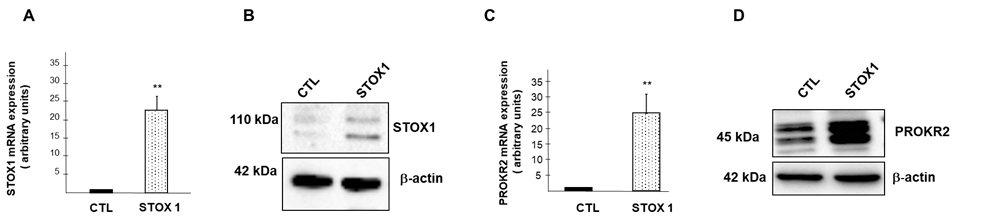


**Figure Supp-3: Validation of the overexpression of STOX1 in JEG-3 trophoblast cells. Panels A and B** report the comparison of the expression of STOX1 mRNA and protein in CTL and STOX1 overexpressing cells, respectively. **Panels C and D** report the comparison of the expression of PROKR2 mRNA and protein in CTL and STOX1 overexpressing cells, respectively.

**Fig. Supp-4**

**
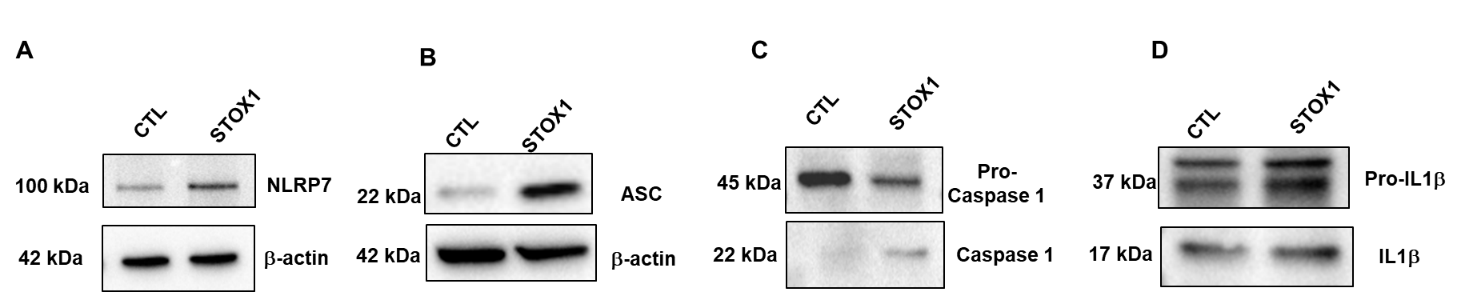
**

**Figure Supp-4: Consequences of the overexpression of STOX1 on the expression of proteins of the inflammasome machinery. Panels A, B, C & D** report the comparison of the expression of NLRP7, ASC, pro-caspase1, pro-IL1β in CTL and STOX1 overexpressing cells, respectively.

**Material and methods on cellular analyses**

**Preparation of HUVEC cells**.

Primary HUVEC were isolated from umbilical vein [[1](#_ENREF_1)] by treatment with collagenase (0.1% from Clostridium histolyticum) for 20 min at 37°C. They were grown to reach confluence in M199 medium supplemented with 10% FBS, 2% Low Serum Growth Supplement LSGS (Cascade Biologics, USA) as previously described [[2](#_ENREF_2)]. The 80% confluent HUVECs (passages 2 or 4) were used for most of the experiments.

**Western Blot analysis**

Proteins were extracted from JEG-3-STOX1 and HUVEC using RIPA lysis buffer and processed as previously described. The blotted membranes were blocked with 5% skim milk in PBS containing 1% Tween-20 (for STOX1, PROKR2, ASC, pro-caspase, caspase, NLRP7 and β-actin membranes) or 2% Bovine serum albumin in Tris-buffered saline (TBS) containing 0.1% Tween 20 (for P38, pro-IL-β and IL-1β) for 1 hour. After washing with 1x TBS-T 1%, the membranes were then incubated overnight at 4°C with the following primary antibodies: Il-1β (1/200, Santa Cruz), P38 (1/1000, Cell Signaling Technology), STOX 1 (1µg/ml, Covalab), PROKR2 (0.5 µg/ml Covalab), NLRP7 (5.6 µg/mL, Covalab, Bron, France), PCNA (0.1 µg/mL, Becton Dickinson), anti-CD31 (4 µg/mL, Abcam). To standardize for sample loading, the blots were subsequently stripped using a commercially available kit, following the manufacturer's instructions (Mild Reblot; Millipore), and re-probed with an anti-β-actin antibody (1/8000, Sigma-Aldrich) as an internal control for total protein loading.

**Preparation of conditioned media from JEG-3-STOX1 cells**.

STOX1 transfected JEG3 (JEG3-STOX1) cells were treated with or without PROKR2 antagonist (1µM) for 24h. Media were collected, centrifuged at 1000 rpm / 5 min to remove all debris and then concentrated approximately 25-fold using Centriprep (Centrifugal Filter Units with an Ultracel 9k membrane (Pierce, Thermo Scientific). The concentrated media was diluted 10 times in the HUVEC culture media.

**Immunofluorescence of HUVEC**

HUVEC were seeded on glass cover slips, grown to 80% confluency and treated with naive media or CM media collected from JEG3-STOX1 treated or not with PKRA. They were the fixed and incubated with anti-VE-cadherin, an in house antibody (1/1000) [[3](#_ENREF_3)]. The cells were then washed with PBS three times. Donkey anti-rabbit IgG secondary antibody (Alexa Fluor 488 conjugate, Jackson Immuno research Inc, USA) was used at 1/200 dilution. Images were taken by Zeiss fluorescent microscopy.

**Wound healing/Migration assay of HUVEC**

Wound healing/migration assay was performed to examine the effect of CM collected from JEG-3 STOX1 cells that were treated or not with PKRA, on migratory potential of the HUVEC. Briefly, cultured HUVEC were seeded at 3x10**^5^** cells/well into 24-well plates. At confluence, the cells were scrapped with a sterile tip to create an artificial wound and incubated with the treatment of interest. The wounds were allowed to heal by the migratory potential of HUVEC for the next 24 h. Photographs were taken at T0 and T24h. The size of the wound was measured on photographs taken from three separate experiments using ImageJ (Wayne Rasband, USA). The results are reported as percentage of wound closure/migratory potential of HUVEC following 24 h post treatment.

**References**

1. **Barbieri B, Balconi G, Dejana E, Donati MB.** Evidence that vascular endothelial cells can induce the retraction of fibrin clots. *Proc Soc Exp Biol Med*. 1981; 168: 204-7.

2. **Brouillet S, Hoffmann P, Benharouga M, Salomon A, Schaal JP, Feige JJ, Alfaidy N.** Molecular characterization of EG-VEGF-mediated angiogenesis: differential effects on microvascular and macrovascular endothelial cells. *Mol Biol Cell*. 2010; 21: 2832-43.

3. **Sidibe A, Mannic T, Arboleas M, Subileau M, Gulino-Debrac D, Bouillet L, Jan M, Vandhuick T, Le Loet X, Vittecoq O, Vilgrain I.** Soluble VE-cadherin in rheumatoid arthritis patients correlates with disease activity: evidence for tumor necrosis factor alpha-induced VE-cadherin cleavage. *Arthritis and rheumatism*. 2012; 64: 77-87.
